# Video-Assisted Mastectomy with Immediate Breast Reconstruction: First Clinical Experience and Outcomes in an Eastern European Medical Center

**DOI:** 10.3390/cancers17132267

**Published:** 2025-07-07

**Authors:** Adrian Daniel Tulin, Daniela-Elena Ion, Adelaida Avino, Daniela-Elena Gheoca-Mutu, Abdalah Abu-Baker, Andrada-Elena Țigăran, Teodora Timofan, Ileana Ostafi, Cristian Radu Jecan, Laura Răducu

**Affiliations:** 1 Discipline of Anatomy, ‘Carol Davila’ University of Medicine and Pharmacy, 020021 Bucharest, Romania; adrian.tulin@umfcd.ro (A.D.T.); daniela-elena.mutu@umfcd.ro (D.-E.G.-M.); ileana.dima@rez.umfcd.ro (I.O.); 2 Department of General Surgery, ‘Prof. Dr Agrippa Ionescu’ Clinical Emergency Hospital, 011356 Bucharest, Romania; 3 Discipline of Plastic Surgery, ‘Carol Davila’ University of Medicine and Pharmacy, 020021 Bucharest, Romania; abdalah.abu-baker@drd.umfcd.ro (A.A.-B.); andrada-elena.tigaran@rez.umfcd.ro (A.-E.Ț.); teodora.peligrad@rez.umfcd.ro (T.T.); cristian.jecan@umfcd.ro (C.R.J.); laura.raducu@umfcd.ro (L.R.); 4 Department of Plastic and Reconstructive Surgery, ‘Prof. Dr Agrippa Ionescu’ Clinical Emergency Hospital, 011356 Bucharest, Romania

**Keywords:** breast cancer, nipple-sparing mastectomy, video-assisted mastectomy, endoscopic mastectomy, prophylactic mastectomy, risk-reducing surgery, direct-to-implant reconstruction, prepectoral breast reconstruction

## Abstract

Background/Objectives: The aim of this case series is to evaluate the outcomes and safety of video-assisted mastectomy, illustrating the harmonious collaboration of oncologic and plastic surgery. This novel minimally invasive technique allows immediate prosthetic reconstruction and represents a cost-effective alternative to robotic breast surgery. Methods: Video-assisted, single-port nipple-sparing mastectomies were performed in patients with small to medium-sized breasts, followed by immediate direct-to-implant reconstruction with either prepectoral or dual plane implant placement. The patients’ electronic medical records were analyzed, including demographic characteristics, operative times and histopathology reports. Results: A total of 18 patients underwent successful video-assisted mastectomy, without conversion to traditional open procedure. Fifteen of the operations were risk-reducing mastectomies. Twelve patients had complementary procedures performed concurrently on the previously operated contralateral breast (delayed reconstruction/expander-to-implant exchange). Moreover, three patients benefited from additional minimally invasive techniques during the same surgery (prophylactic laparoscopic hysterectomy). Immediate breast reconstruction with polyurethane or microtextured breast implants up to 450 cc was performed, with satisfactory aesthetic outcomes and no cancer recurrences at 6 to 12 months postoperative follow-up. Early complications included transient hypercapnia, areolar congestion and cellulitis. No skin necrosis or implant-related complications were reported. The most frequently encountered late issues were contour irregularities. Conclusions: Video-assisted mastectomy facilitates the safe removal of proven pathologic or healthy breast tissue with minimal damage to the breast’s skin envelope, facilitating single-stage breast reconstruction.

## Simple Summary

Minimally invasive techniques are acclaimed in all surgical fields for ensuring minimal tissue trauma and blood loss leading to faster recovery and higher patient satisfaction. In risk-reducing (prophylactic) as well as oncologic breast surgery, these objectives can be achieved through video-assisted endoscopic mastectomy followed by immediate breast reconstruction.

## 1. Introduction

Breast reconstruction has had a monumental impact on cancer survivors’ psychological recovery, boosting their self-esteem and aiding their reintegration into society [1,2]. These patients are faced with more subtle aspects that may not be obvious to other people and are sometimes minimalized even by the reconstructive surgeons or the patients themselves, such as lack of the nipple, insensate breasts or large scars. These affect their quality of life and may also represent a constant reminder of an unfortunate event, fueling their anxiety towards the future [3,4].

Nipple reconstruction and breast neurotization have been viewed by plastic surgeons as the pinnacle of their reconstructive efforts and a challenge to further improve their patients’ quality of life, leading to numerous technical innovations all over the world during the past decades [5,6]. Meanwhile, the aesthetic outcomes of the reconstruction and the appearance of the scar have been progressively improved through the introduction of less invasive techniques. Due to the superficial nature of the breast, the absence of a natural cavity for endoscopic exploration, the frequent need to remove the skin itself, particularly the nipple–areolar complex (NAC), and the relatively low morbidity of conventional breast surgery, the evolution of breast surgery towards minimal-access techniques has been more circuitous [4,7,8,9,10]. With each step guided by the urge to balance oncologic safety and aesthetics, tumor ablation has gradually advanced from radical to modified mastectomy, from breast conserving surgery (BCS) to oncoplastic surgery (both overshadowed by the unpredictable detrimental effects of radiotherapy on the appearance of the remaining breast) and finally to skin- and nipple-sparing mastectomy (NSM) with immediate reconstruction, minimizing the psychological impact of the procedure [2,3].

Endoscopy-assisted nipple-sparing mastectomy (NSM) was introduced in 2002 in Japan and shortly thereafter in China, aiming to optimize cosmetic outcomes through small, hidden incisions (periareolar, axillary, umbilical, inframammary fold) as well as to diminish the need for skin retraction when the breast is accessed through remote sites [11,12,13,14]. Nowadays minimal-access techniques can be performed through a sole incision by implementing single-port technology, which has concurrently decreased the incidence of skin flap and NAC necrosis [4,13,15,16,17,18,19,20].

Direct visualization of anatomical structures such as the intercostal perforator branches facilitates their protection and lowers intraoperative blood loss, while insufflation assists in the creation of well-defined dissection planes for secure mammary gland removal with minimal trauma to the preserved tissues, contributing to increased trophism, unaltered color of the NAC, shorter hospital stays and quicker recovery [4,21,22,23]. Most complications commonly associated with open breast surgery and reconstruction (seroma, hematoma, dehiscence and prosthetic exposure) are avoided through the creation of superior-quality skin flaps under endoscopic guidance supplemented by limited risk of prosthesis exposure or infection because of distant incision placement [24,25,26,27,28]. These advantages allow safe, single-stage implant breast reconstruction in the prepectoral plane, which offers the benefit of limited shoulder stiffness and postoperative pain as well as nullifying the risk of animation deformity typical for retropectoral placement, in addition to decreasing the chances of fluid accumulation with subsequent breast deformity or infection [24,25,26,29]. When supplemented by preoperarative skin envelope assessment (through digital mammography, as proposed by Rancati, who created the breast tissue coverage classification—BTCC—to identify patients at risk for flap ischemia and necrosis) and careful selection of implant size and type, such as microtextured or polyurethane-coated devices, prepectoral implant placement can maintain a more natural breast contour with lower rates of capsular contracture [17,25,29,30,31].

This minimally invasive technique perfectly aligns with the goals of 21st-century reconstructive breast surgery, which extend beyond securing oncological safety towards ensuring an adequate quality of life to breast cancer survivors through the reconstruction of an aesthetically pleasing breast that does not compromise the function or appearance of the surrounding structures [1,22,24,32,33,34].

VAM may also be offered as a comparably minimally invasive procedure to patients who reject BCS or the subsequent radiotherapy or have small breasts in which oncoplastic resection is incompatible with maintaining symmetry [12]. Some studies also include known contraindications to BCS such as the presence of microcalcifications, multicentric or multifocal tumors and extensive intraductal spread among the indications of endoscopic-assisted mastectomy [12,15,35].

Indications for VAM include risk-reducing (prophylactic) mastectomy (BRCA1/2 mutation, high-risk lesions such as lobular carcinoma in situ, atypical ductal hyperplasia, high-risk patients requesting risk-reducing mastectomy after counseling), early-stage breast cancer (T1 or T2, stage II or below, no evidence of distant metastasis), extensive ductal carcinoma in situ, no evidence of chest wall, pectoralis muscle, skin (ulceration, infiltration) or nipple involvement (hemorrhage, invagination, discharge, Paget’s disease) and tumor location more than 2 cm away from the NAC [8,21,22,23,24,36,37]. Other factors precluding the application of minimally invasive NSM are centrally located tumors, severe lymphovascular invasion and inflammatory breast cancer, as well as obesity, large breast volume/cup > C and severe ptosis, which would require additional mammoplasty for satisfactory cosmetic outcomes [8,21,22,23,24].

We illustrate our experience with video-assisted mastectomy with a case series including both risk-reducing mastectomy (RRM) and therapeutic video-assisted mastectomy (TM). Being aware of the oncologic consequences of incomplete tumor removal, we initially applied this novel technique to patients having previous history of contralateral breast cancer and mastectomy who were scheduled for delayed breast reconstruction and expressed their desire to undergo risk-reducing mastectomy with immediate reconstruction instead of symmetrization of the healthy breast. Collaborating with an oncologic surgeon endowed with ample experience in laparoscopic surgery ensured swift overcoming of the learning curve and confirmed this procedure’s capacity to create adequate skin flaps for immediate prepectoral implant-based reconstruction, foregoing the need for subpectoral placement with its complications such as shoulder stiffness and animation deformity [25,26]. The enhanced precision of the dissection allowed not only the preservation of the circummammary ligaments and native breast shape with high implant stability in the absence of acellular dermal matrix or synthetic mesh coverage, especially when using polyurethane-coated implants, but also aided in attaining complete gland excision.

The aim of the study is to prove the feasibility and safety of video-assisted mastectomy and immediate breast reconstruction as a minimally invasive and aesthetically pleasing alternative to conventional nipple-sparing mastectomy.

## 2. Materials and Methods

This was a single-center case series conducted in the Department of Plastic and Reconstructive Surgery of the ‘Prof. Dr Agrippa Ionescu’ Clinical Emergency Hospital in Bucharest, Romania, between 1 September 2023 and 31 April 2024.

The patients included in the study received approval from a multidisciplinary tumor board and were selected based on the following inclusion criteria:

1. Therapeutic VAM (TM): breast cancer patients with histologically confirmed diagnosis prepared to undergo oncologic resection and the following tumor characteristics: T2 or below, stage I or II, absence of NAC, skin or pectoralis major invasion according to preoperative imaging and clinical examination, more than 2 cm distance from tumor to NAC, absence of lymphovascular spread, immunohistochemistry phenotype other than triple negative;
2. Risk-reducing VAM (RRM): breast cancer patients with contralateral mastectomy ± reconstruction with no evidence of locoregional or distant recurrence willing to undergo removal of the remaining breast for the following reasons: the presence of BRCA1/2 mutations, familial history of breast or gynecological cancer, young age at initial diagnosis.

Prior to surgery, all the patients signed an informed consent form after receiving information regarding the advantages and potential risks associated with the procedure.

**Figure 1:**
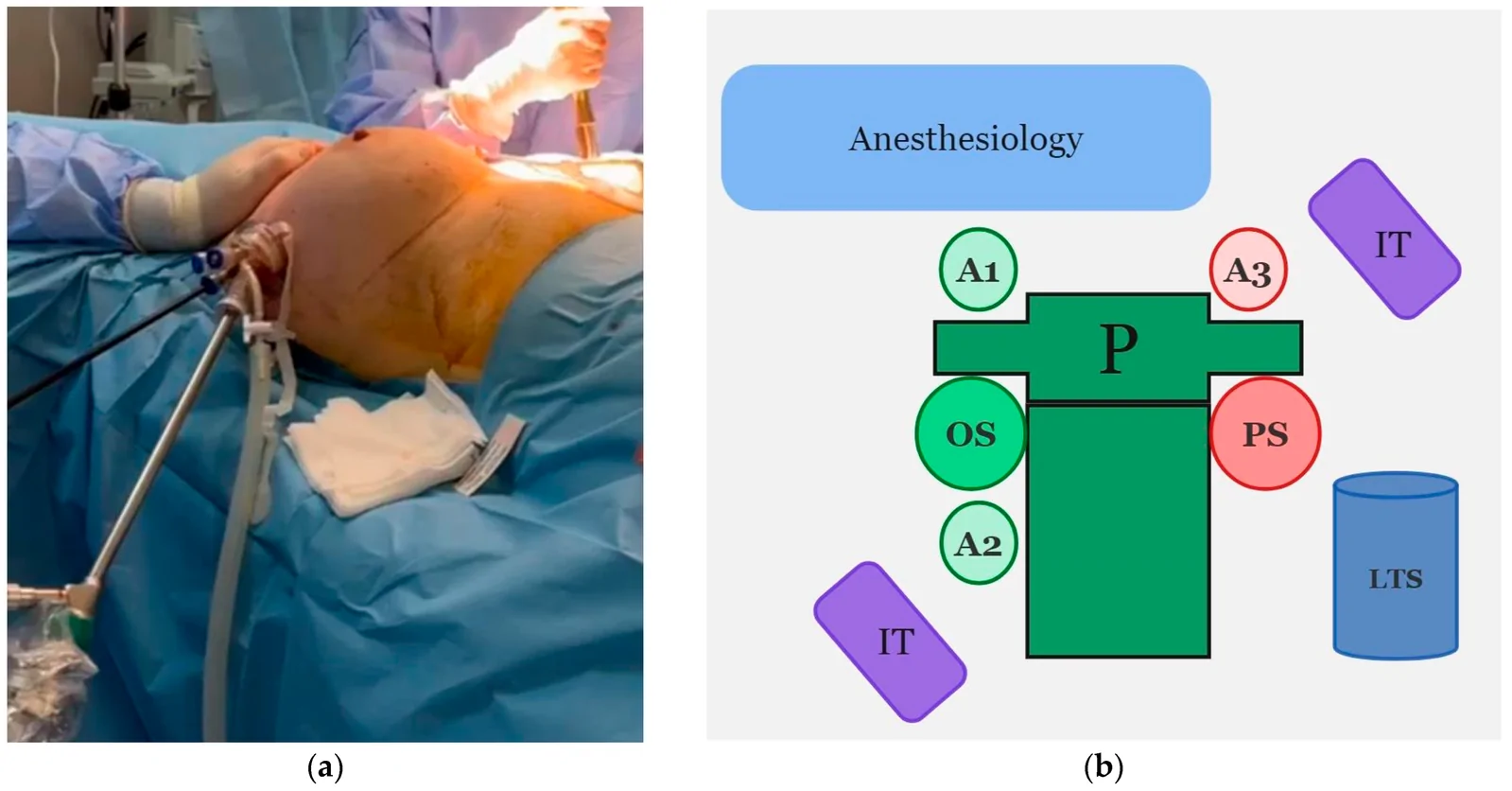
(**a**) Real-life setting (VAM—right breast, reconstruction—left breast, patient’s head upward): OS’s instrument and A2’s camera entering the single-port piece visible on the right side of the patient, A3 visible on the opposite side; (**b**) operative theatre setting for bilateral surgery. P: patient, LTS: laparoscopy tower system, OS: oncologic surgeon, PS: plastic surgeon, A1, A2, A3: assistants, IT: instrument table.

Preoperative markings were drawn in advance with the patient in standing position. Prophylactic i.v. antibiotics (2 g of Cefazolin) were administered prior to the intervention, followed by 3 additional 1 g doses administered every 12 h postoperatively. After intubation, the patient was positioned supine with both arms abducted at 90°, carefully disinfected with betadine and draped. The operative theatre setting for bilateral surgery is illustrated in Figure 1.

The same trained oncologic surgeon performed the VAM in all the patients, while the reconstructive procedure was executed by one of two plastic surgeons. The duration of the main operative steps was recorded to assess efficacy and delineate the learning curve, as well as the total operative time. Operative times were recorded as follows:

1. Superficial plane dissection time;
2. Prepectoral plane dissection time;
3. Total mastectomy time: from skin incision to mastectomy specimen removal and hemostasis, including the previous two times;
4. Reconstructive time: from breast pocket measurement to skin closure;
5. Total operative time: from incision to skin closure.

A linear incision measuring 2.5 cm initially was made in the axilla, placed parallel to minimal skin tension lines and abutting the anterior axillary line. Sentinel lymph node dissection could also be performed at this point to ensure that frozen section evaluation was complete by the time the mastectomy had ended. If necessary, axillary lymph node dissection can further be accomplished after VAM is completed, to allow further incision enlargement without the risk of air leakage.

**Figure 2:**
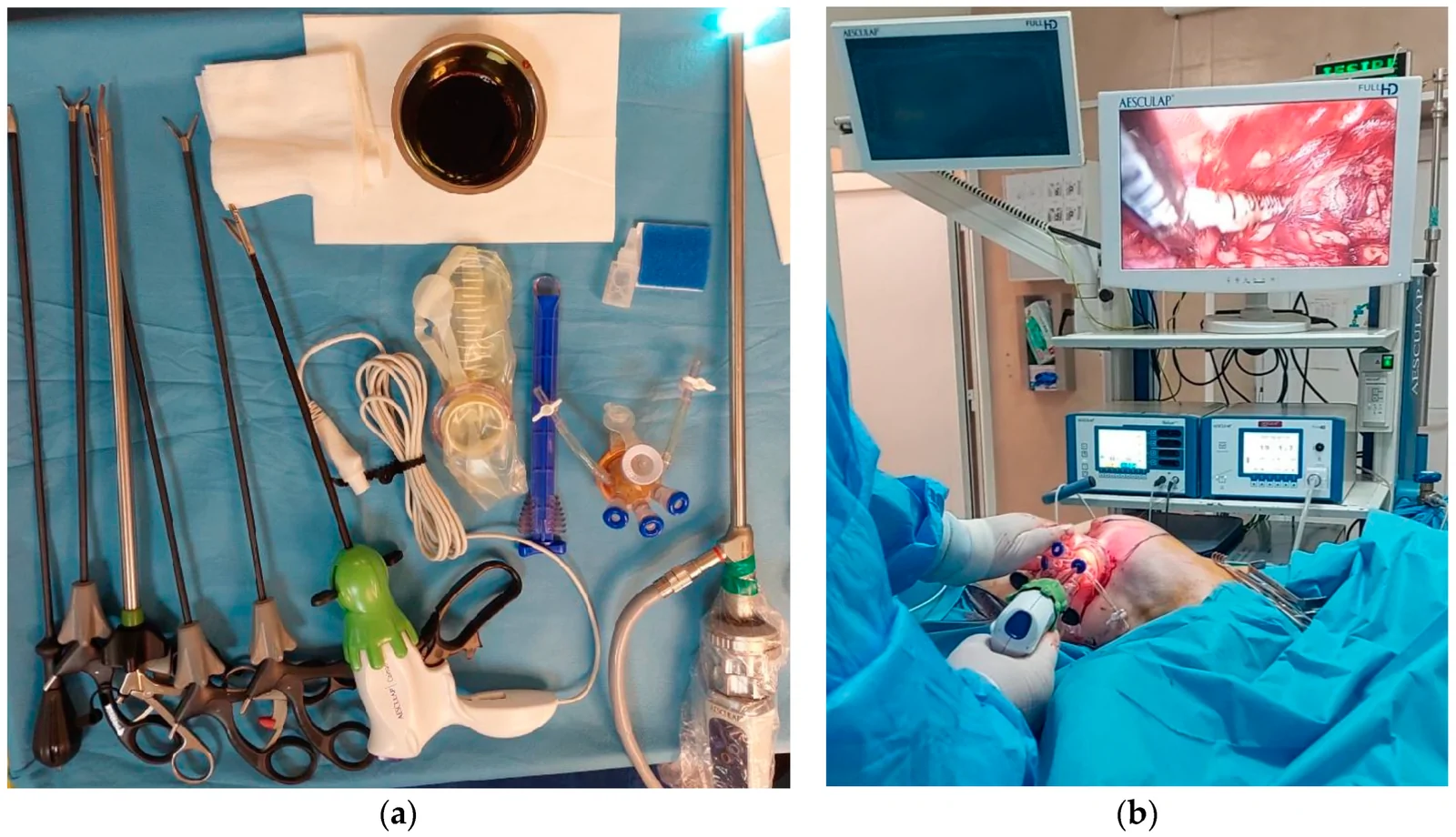
(**a**) Instruments used in the mastectomy phase (from left to right): laparoscopic atraumatic grasping instruments (×5), Caiman vessel sealer and cutter, wound protector, single-port trocar, laparoscopic camera; (**b**) intraoperative setting—unilateral surgery (VAM after sentinel lymph node excision).

Dissection began under direct vision until the superficial layer of the breast fascia and the lateral border of the pectoralis major muscle were identified, then it proceeded to 2–3 cm in the pre- and retromammary planes, anticipating the relative difficulty of endoscopic dissection around the entry point of the trocars. Next, a single-port triple+ trocar piece was inserted, followed by CO_2_ insufflation using a constant pressure of 8 mmHg. Myorelaxants were administered by the anesthesiologist before the air cavity was created, and CO_2_ pressure was constantly monitored throughout the entire endoscopic stage of the procedure. Video-assisted dissection started in the superficial plane, the mammary gland being dissociated from the subcutaneous tissue beginning from the upper outer quadrant and advancing clockwise to the entire surface of the gland. The NAC was approached between the upper and the inferior inner quadrants with sharp dissection, using minimal coagulation in this area. Tissue samples from its base were collected and analyzed on frozen sections, in addition to tumor resection margins, whenever TM was performed. The thickness of the skin flaps was maintained by severing Cooper’s ligaments at constant lengths under endoscopic visualization, as well as constant assessment through tactile feedback by the main operator, while the assistant aided in preserving the breast footprint by monitoring transillumination against the preoperative markings and providing dynamic feedback by tapping along them when the main operator employed both hands in the dissection. After superficial plane dissection was completed, prepectoral dissection was initiated, advancing in the same manner, without lifting the fascia of the pectoralis major. In this case, the endoscope doubled as a retractor, with occasional assistance from an atraumatic grasping instrument. All the instruments typically used in this phase can be observed in Figure 2. The incision can be enlarged to allow the extraction of the gland, especially for medium-sized and higher-density breasts. The site of the NAC and the tail of Spence were marked, and the resected specimen was sent for histopathologic examination. The results of frozen section assessment from the NAC and any sentinel lymph nodes removed were ascertained before proceeding further.

**Figure 3:**
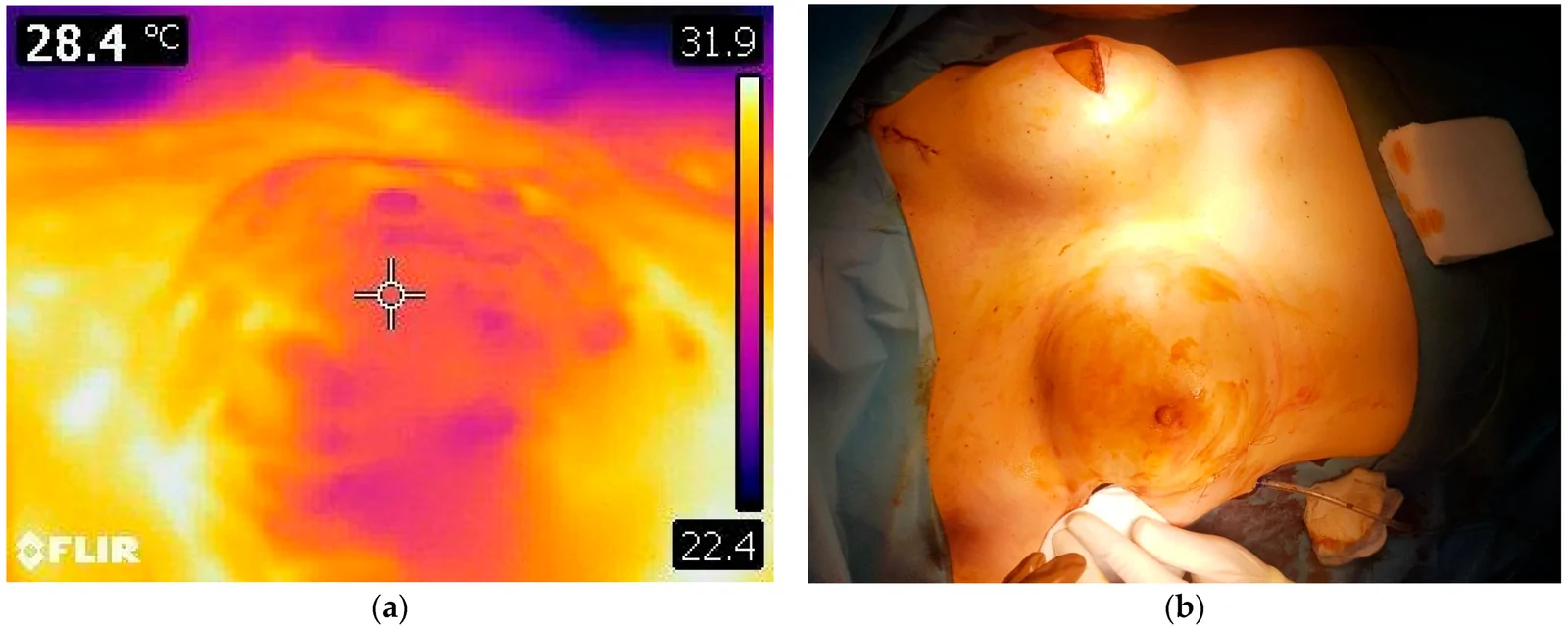
(**a**) Skin perfusion evaluation using infrared thermography after VAM and implant placement (good perfusion: 27.9 °C on nipple, 28.4 °C surrounding skin flap); (**b**) symmetry assessment after implant insertion (right breast—VAM and implant reconstruction, left breast—expander replacement with implant).

The reconstructive stage was initiated after final inspection and hemostasis of the breast pocket. Implant choice was guided by the intraoperative measurement of the breast pocket, as well as the thickness of the flaps and the volume of the contralateral breast, guided by the preoperative estimations. Flushing of the cavity with betadine and drain tube placement along the lower pole were performed in advance, and the elected implant itself was washed with betadine solution. Insertion was performed with the aid of the sterile funnel enclosed with the implant, followed by skin redraping, elevation of the patient to ascertain symmetry (after contralateral implant or sizer insertion in case of delayed reconstruction; see Figure 3) and double-layer closure with absorbable monofilament 2-0 sutures in the subcutaneous plane and removable intradermal 3-0 polypropylene sutures.

In most cases, delayed or second-stage reconstruction on the contralateral breast was carried out concurrently with the VAM. However, some patients underwent additional axillary (sentinel lymph node biopsy) or gynecological procedures (laparoscopic prophylactic hysterectomy and bilateral salpingo-oophorectomy) during the same anesthetic session, resulting in great variation of the total operative time.

Supplementary intraoperative records included the final length of the incision, volume of the mastectomy specimen, breast implant type and volume, CO_2_ insufflation pressure required to maintain an adequate working space and skin flap perfusion, which was assessed using an infrared thermal device (FLIR E50 Handheld Thermal Camera, Teledyne FLIR, Wilsonville, OR, USA), as shown in Figure 3, and classified as good or suboptimal depending on the detection of temperature differences over 2 degrees Celsius between the coolest area on the breast envelope and the average temperature of the adjacent thoracic skin.

Postoperative data focused on pain assessment using the VAS (visual assessment scale), total drainage duration and length of hospital stay, pathologic assessment of the mastectomy specimen, and immediate and delayed complications. All patients wore personally tailored bras starting from the first or second postoperative day. Follow-up visits were scheduled at 3, 6 and 12 months postoperatively, including photographic assessments in addition to patient-reported quality of life evaluation using the BREAST-Q questionnaire and physician-reported aesthetic evaluation using the Harris and Ueda scales [38,39,40].

## 3. Results

### 3.1. Patient Selection

Among the 90 patients proposed for either therapeutic or risk-reducing mastectomy with immediate prosthetic breast reconstruction during the study interval, after evaluation by a multidisciplinary tumor board 72 were excluded due to the following factors:

- Contraindications to NSM: nipple involvement (*n* = 5), tumor location within 2 cm from NAC (*n* = 8), multifocal/multicentric lesions (*n* = 1), subareolar microcalcifications (*n* = 3), lymphovascular spreading (*n* = 4), large tumor (*n* = 3);
- Proximity of the tumor to the skin surface or pectoralis muscle (*n* = 7), axillary lymph node involvement (*n* = 10), triple negative phenotype (*n* = 4);
- Recurrent or metastatic breast cancer (*n* = 2);
- Large breast size requiring implant volumes over 500 cc (*n* = 7);
- Ptosis grade >= 2 according to Regnault’s classification ± BMI > 30, requiring skin flap reduction (*n* = 8);
- Poor breast coverage (type 1) according to the BTCC classification, as measured by digital mammography (*n* = 4);
- Severe comorbid conditions (*n* = 1);
- Significant psychological or psychiatric conditions (*n* = 2);
- Autologous breast reconstruction option (*n* = 3).

**Figure 4:**
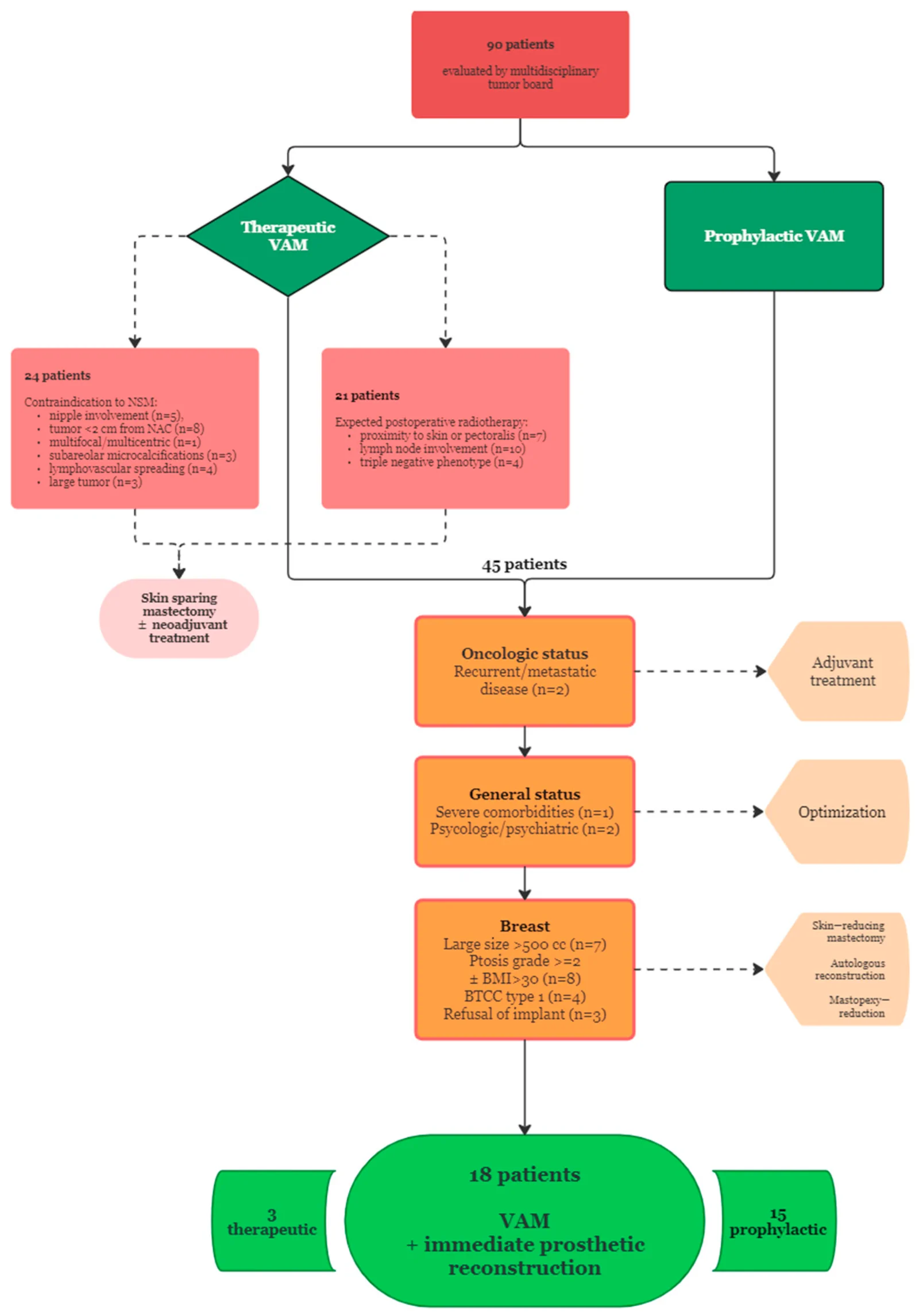
Patient selection flowchart.

Consequently, a total of 18 patients underwent VAM during the aforementioned time range (15 RRM, 3 TM). The selection process is delineated in Figure 4.

### 3.2. Demographic and Oncologic Characteristics

The patients in the case series were mainly premenopausal women (61.11%, with a mean age of 47.56 years (37–77) and mean BMI of 25.13 (20.2–29.62), with small to medium-sized breasts (estimated volume ranging from 154 to 405 mL, mean: 283.67 mL), grade 1 ptosis according to Regnault’s classification (55.56%) and type 3—good breast coverage according to the BTCC classification (61.11%). In total, 27,78% of patients were active smokers.

TM was performed in three patients, one of whom also had a modified radical mastectomy on the contralateral breast and desired simultaneous bilateral reconstruction. A total of five patients required contralateral reconstruction after previous mastectomy performed in other hospitals (four with RRM and one with TM), whereas seven patients undergoing RRM had tissue expanders (inserted in our hospital at the time of the mastectomy), which were replaced with implants during the same surgery. No procedures were carried out on the contralateral breast in six patients: two with TM and four who had already had implant-based breast reconstruction on the contralateral breast but desired RRM on the other breast that had previously been symmetrized.

All the patients in our study had previously been diagnosed with breast cancer, with an average interval of almost 2 years between their first mastectomy and VAM, and seven of them presented BRCA1/2 mutations. Invasive ductal carcinoma was the predominant cancer type (66.67%) and most tumors had an intermediate grade (G2—55.56%), stage II being the most prevalent at diagnosis (38.89%). Most of the patients underwent either chemotherapy or radiotherapy (72.22% and 55.56%, respectively) before they presented for RRM. The oncologic characteristics are summarized in Table 1.

### 3.3. Surgery

**Figure 5:**
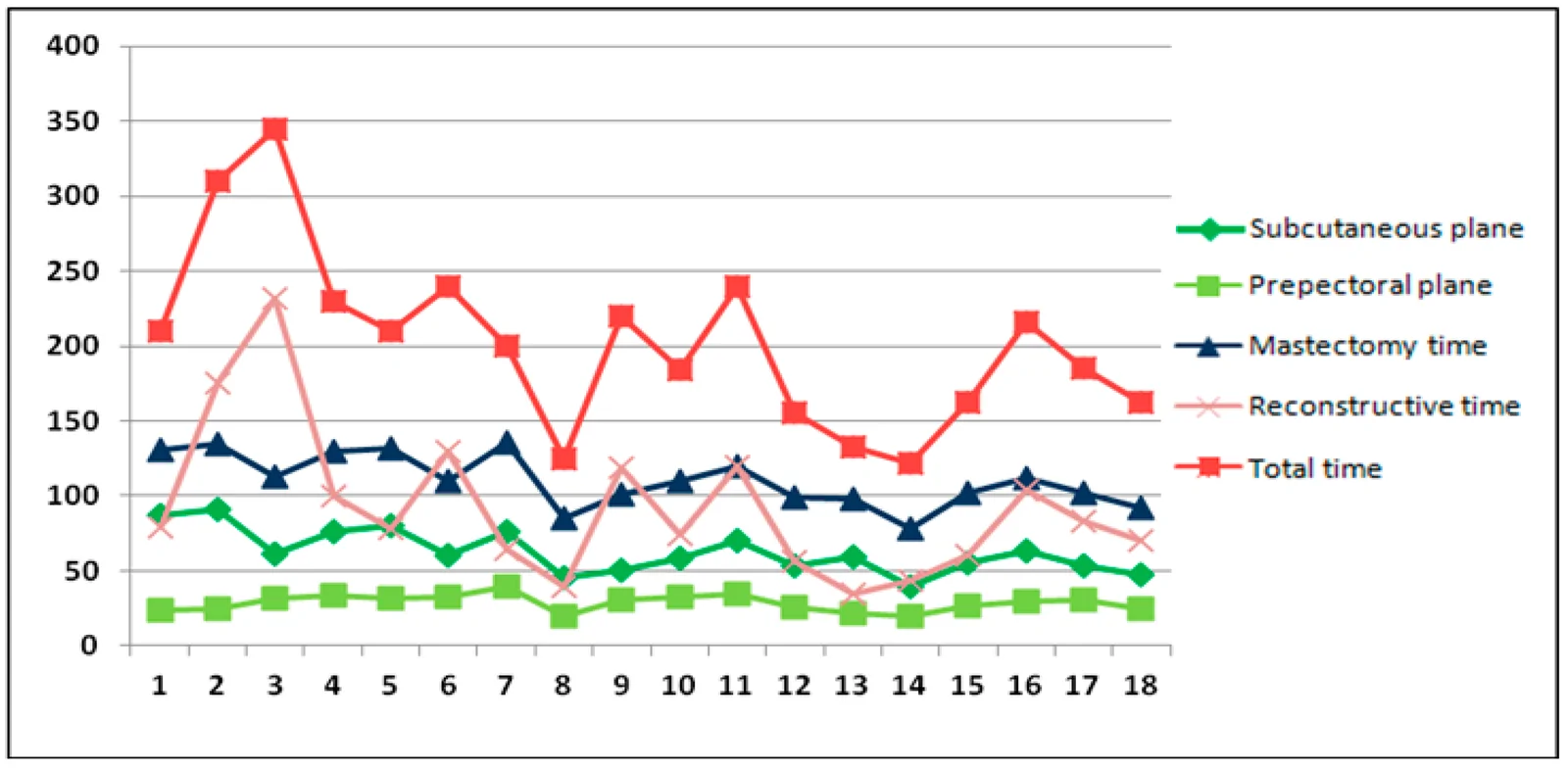
Duration of the operations (minutes): Total time = Reconstruction + Mastectomy time (including Subcutaneous and Prepectoral dissection times supplemented by initial dissection ± sentinel lymph node biopsy and endoscopic setup time). Note the decreasing trend of the stages of VAM and the variability of reconstructive time, especially the peak represented by contralateral latissimus dorsi flap reconstruction performed in patient 3.

The duration corresponding to each stage of the procedure and its evolution across time (from patient 1 to patient 18) is illustrated in Figure 5. For the first three cases, the glandular–pectoral interface was dissected using the retraction method rather than insufflation. CO_2_ pressures were maintained between 7 and 12 mmHg (mean: 9).

The procedures performed concomitantly are enumerated in Table 2. The sentinel lymph node biopsies of patients 5 and 15 were performed prior to the endoscopic stage and are included in the mastectomy times. Meanwhile, the gynecological procedure for patients 6, 11 and 18 took place simultaneously with the reconstructive stage and is included therein.

**Figure 6:**
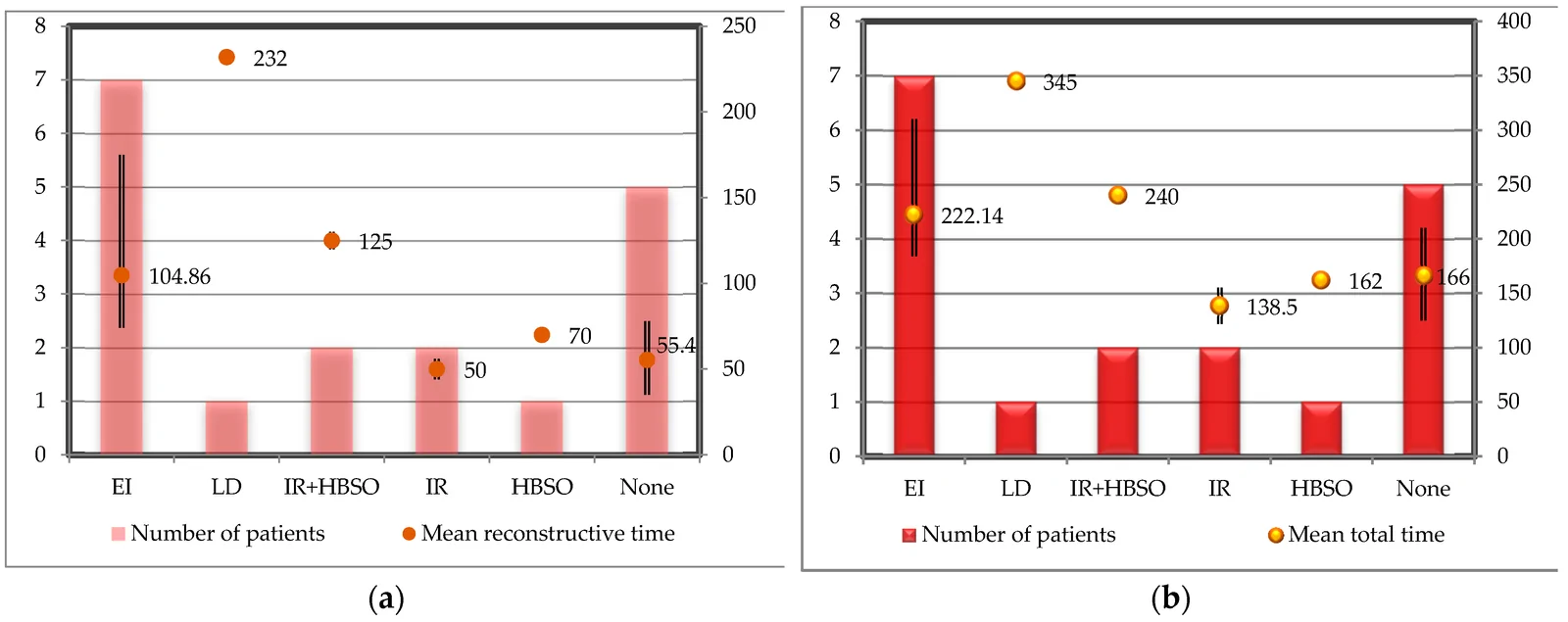
(**a**) Reconstructive time based on concomitant procedure type; (**b**) total operative time based on concomitant procedure type. EI: expander-to-implant exchange; HBSO: hysterectomy and bilateral salpingo-oophorectomy; IR: implant reconstruction; LD: latissimus dorsi and implant reconstruction.

The average mastectomy time was 110.33 min (range: 78–136), the subcutaneous plane dissection lasting longer (62.44 min, range: 40–91) compared to the prepectoral plane dissection (28.89 min, range: 20–40). The mean reconstructive time was 92.38 min, including the procedures performed simultaneously on the patients, most of which were expander replacements with permanent breast implants (7 out of 18 patients) having an average duration of 104.86 min. Only six patients had unilateral breast surgery, with an average reconstructive time of 55.4 min. The reconstructive phase of the procedure lasted from 35 min (unilateral surgery—patient 13) to 232 min (contralateral latissimus dorsi flap and implant reconstruction—patient 4). The addition of prophylactic hysterectomy and bilateral salpingo-oophorectomy resulted in a 75-minute increase of the duration of the second phase of the surgery for bilateral breast reconstruction (two cases) and was limited to a 20-minute increment for unilateral surgery (one case). Summing up, the total duration of the procedure reached an average of 202.72 min (range: 122–345) but only 166 min for unilateral VAM procedures with immediate implant-based reconstruction. The number of patients and mean operative time corresponding to each variation of the procedure are represented in Figure 6.

**Figure 7:**
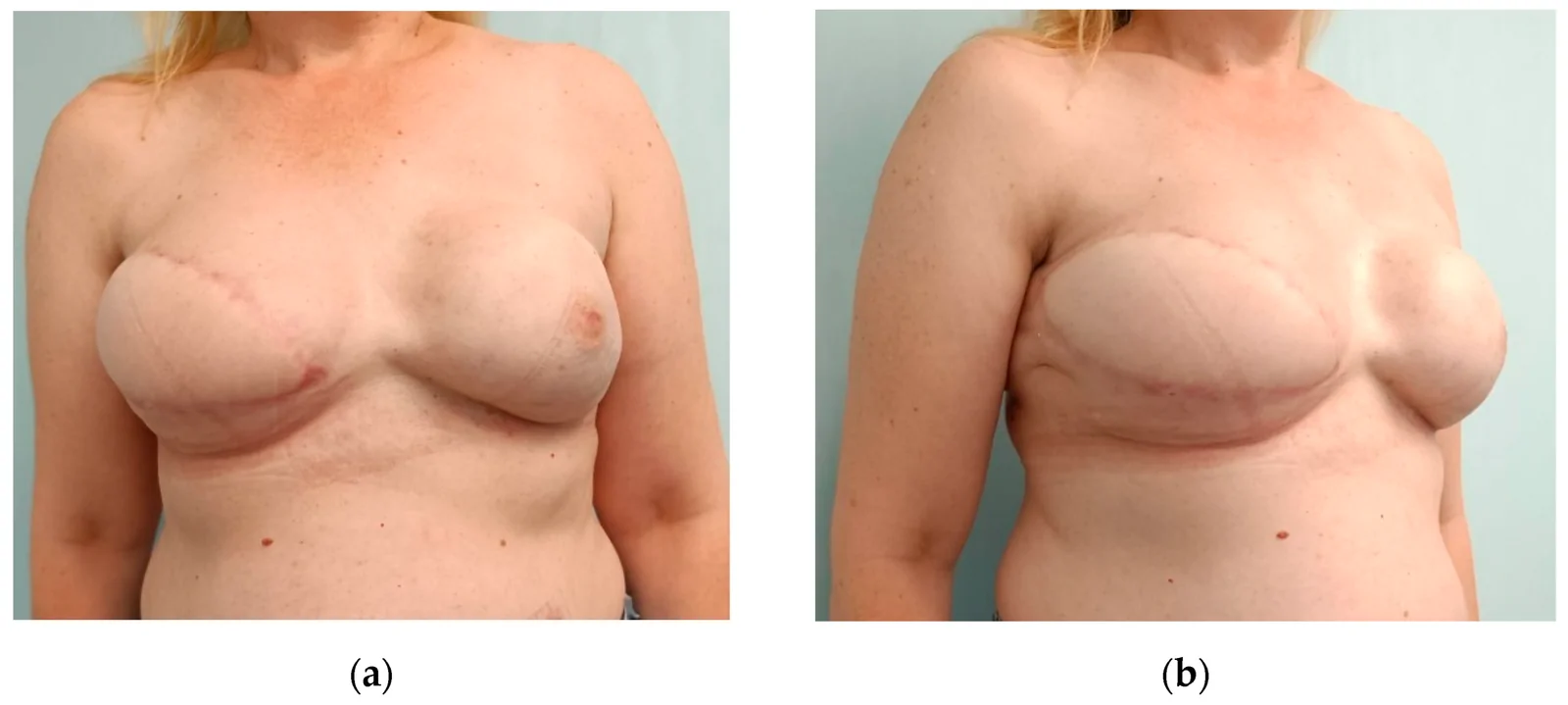
(**a**) VAM with primary implant reconstruction of the left breast and secondary reconstruction of the right breast using latissimus dorsi flap and implant—front view; (**b**) right oblique view. Microtextured implants were used—400 mL for the right breast and 450 mL for the left breast.

Microtextured silicone implants were chosen in six cases, whereas the remaining patients received polyurethane-coated silicone implants, with sizes varying between 195 and 450 mL (mean: 304.72 mL)—see Table 2. The average difference between the chosen implant and estimated volume of the mastectomy specimen was 68.56 mL, while the average difference between the implants selected for the two breasts was 10.31 mL, the largest in the case of the latissimus dorsi reconstruction (50 mL) shown in Figure 7. Incision lengths varied from 2.5 cm to 4.5 cm (mean: 3.19 cm). The skin flaps showed good perfusion after mastectomy in 12 patients, while 6 of them had suboptimal perfusion zones on infrared imaging, predominantly on the NAC and inferior pole, especially in patients with BMI < 25 (4 out of 6).

### 3.4. Postoperative Aspects

The mean postoperative pain score on the VAS scale was 2.22 on the first day after the surgery. The drains were removed when the 24-h quantity dropped below 30 mL, at an average of 8.66 days postoperatively (range 6–17 days). The patients in our case series spent between 4 and 11 days in the hospital (mean 7.44). Those who underwent RRM had a shorter mean hospital stay compared to those referred for TM (6.57 days versus 10.5 days).

The total cost of hospitalization, including the price of the minimally invasive mastectomy and implants, was 4712.02 USD (range: 3160.76–6438 USD), but it was only slightly less expensive for unilateral procedures—4654.82 USD (range: 3483.61–6224.83 USD).

Correlation analyses were performed to assess the relationships between breast volume, hospital stay duration, drainage volume and operative time. A statistically significant moderate positive correlation was found between breast volume and hospital stay (Pearson r = 0.633, *p* = 0.005; Spearman ρ = 0.624, *p* = 0.006), indicating that larger breast volumes are associated with longer hospitalization. Additionally, hospital stay duration was positively correlated with drainage volume (Spearman ρ = 0.641, *p* = 0.004). No significant correlations were observed between breast volume and either drainage volume or operative time, nor between operative time and hospital stay or drainage. Both Pearson and Spearman correlation analyses were conducted to assess the robustness of associations, given the small sample size and potential deviations from normality.

**Figure 8:**
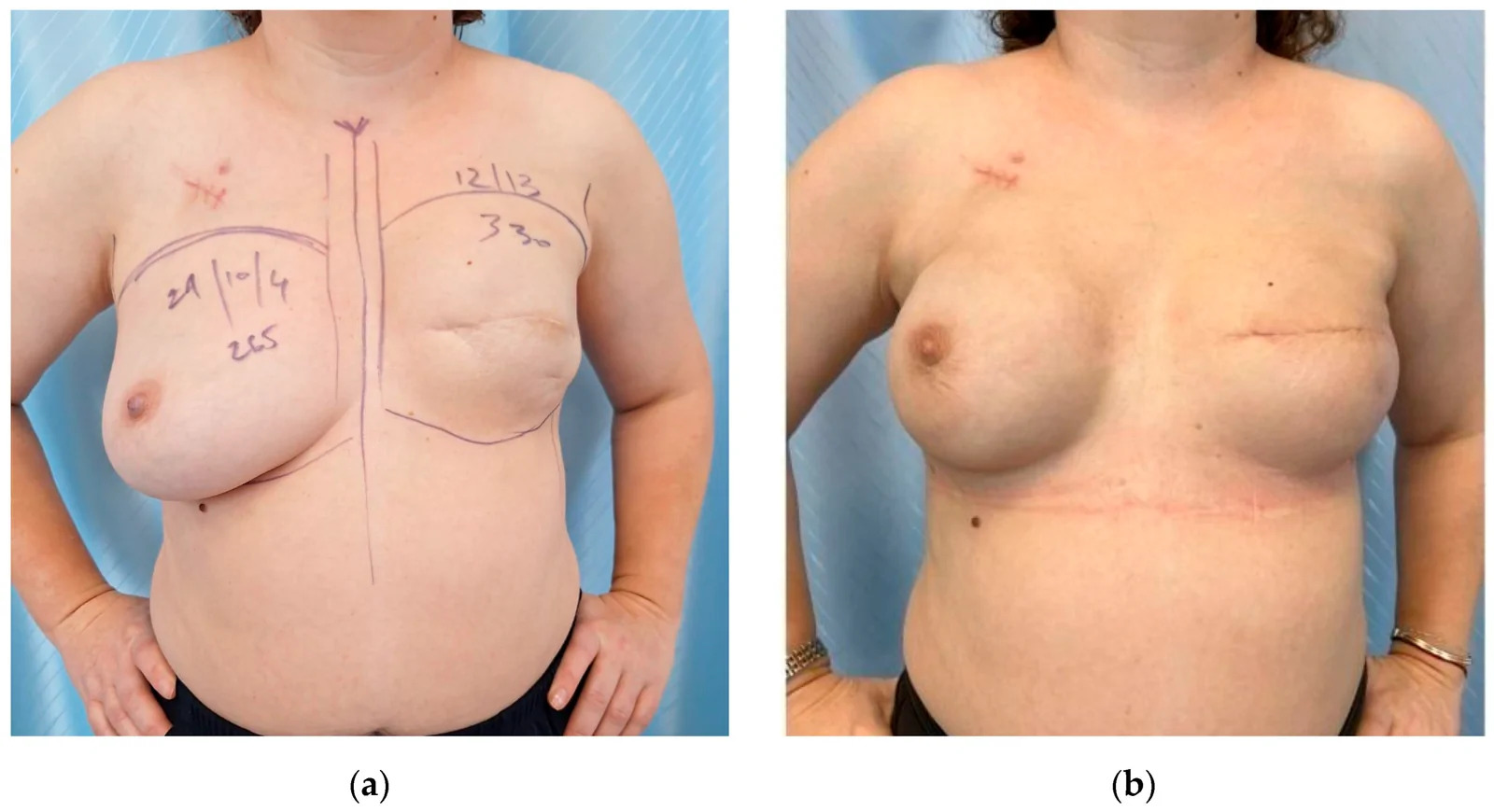
(**a**) Preoperative drawings—left mastectomy with tissue expander placement; (**b**) postoperative results at 6 months—VAM with reconstruction on the right breast with simultaneous contralateral expander-to-implant exchange. Microtextured, 315 mL bilaterally.

**Figure 9:**
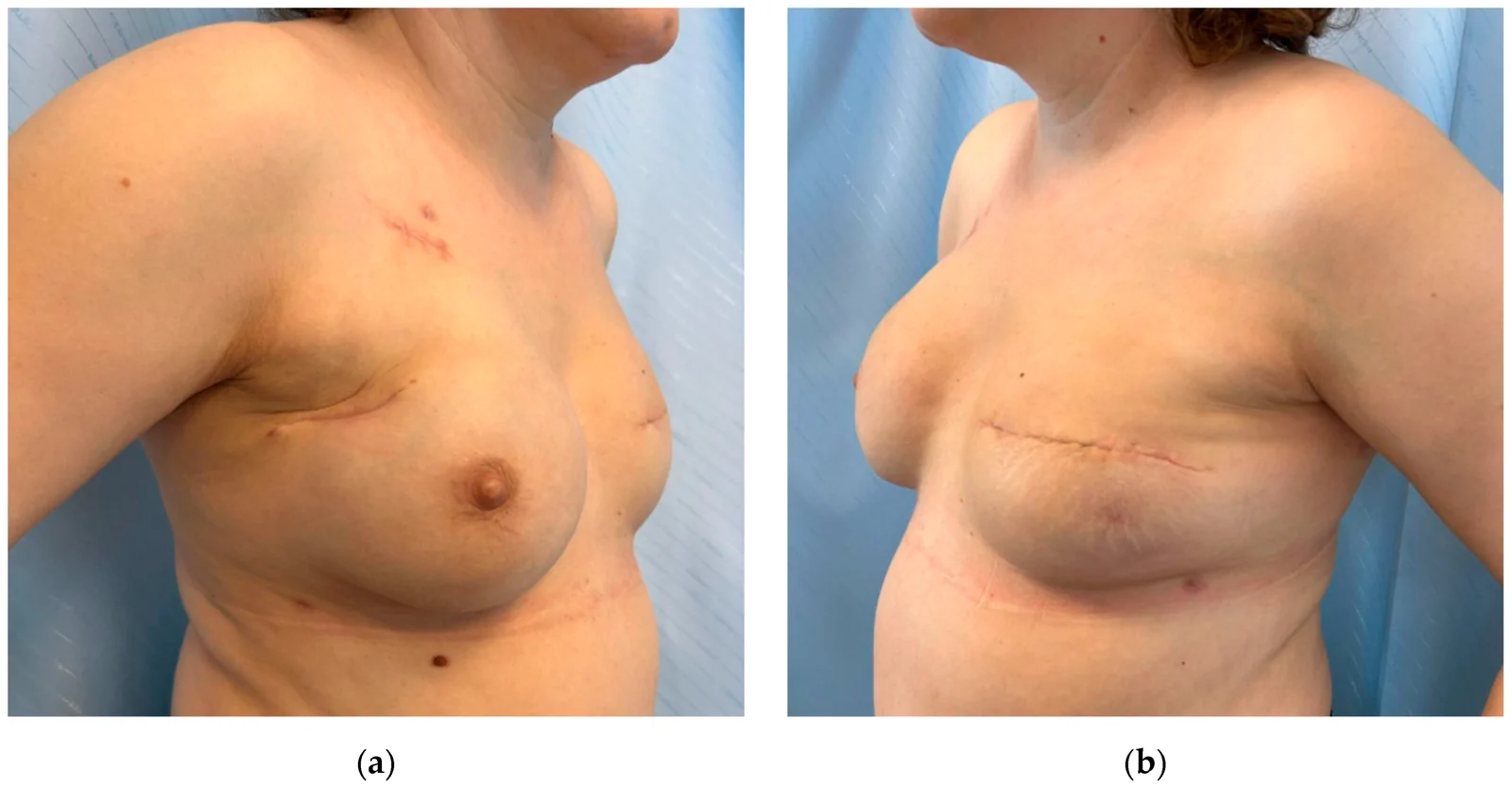
(**a**) Right oblique view at 6 months; (**b**) left oblique view at 6 months. Microtextured 315 mL implants bilaterally.

Regarding complications, two of the patients in our case series, having history of reduction mastopexy on the homolateral breast, developed intraoperative hypercapnia related to the use of insufflation for VAM, which was stabilized by the end of the procedure. Only four patients developed mild postoperative complications: two of the six patients with suboptimal perfusion after implant insertion manifested NAC congestion, which remitted after 3 days of 6-hourly nitroglycerin spray application and wearing a supportive brassiere; another presented wound dehiscence that healed after 3 weeks of conservative management, while the fourth presented with cellulitis 3 weeks after discharge and required i.v. antibiotic treatment. No implant loss due to exposure or implant displacement occurred. Late complications were mainly aesthetic complaints regarding implant border visibility (four cases), as well as contour deformity (two cases) due to unequal subcutaneous fat distribution and asymmetry (three cases); seven of these patients were offered a session of lipofilling to correct these inconveniences. Postoperative results at 6 months are shown in Figure 8 and Figure 9.

During the follow-up period, five patients underwent NAC reconstruction on the contralateral breast at 6 months after the procedure. We consider that the position of the NAC of the breast treated with VAM and reconstruction stabilizes by the 4th to 6th postoperative months and reliably provides a landmark for the position of the NAC on the contralateral breast. However, we decided to delay the reconstruction at least 1 year after the surgery for eight other patients having history of radiotherapy (due to concerns regarding the quality of the skin and capsule, to avoid ischemic and infectious complications, as well as to allow stabilization of the latissimus dorsi flap). The rest of the patients did not require NAC reconstruction (already completed/native NAC present) but were informed regarding the possibility of NAC repositioning for future symmetrization.

No local, regional or distant recurrences were detected in our case series during the follow-up period, but the follow-up time is limited so the results are preliminary. All patients who underwent TM had clear resection margins on final histopathology reports and at least 3 mm from the tumor to the closest resection margin. The rest of the resected specimens showed benign modifications such as fibrocystic disease (50.00%) and fibrosis with adenomatous lesions (33.33%).

**Figure 10:**
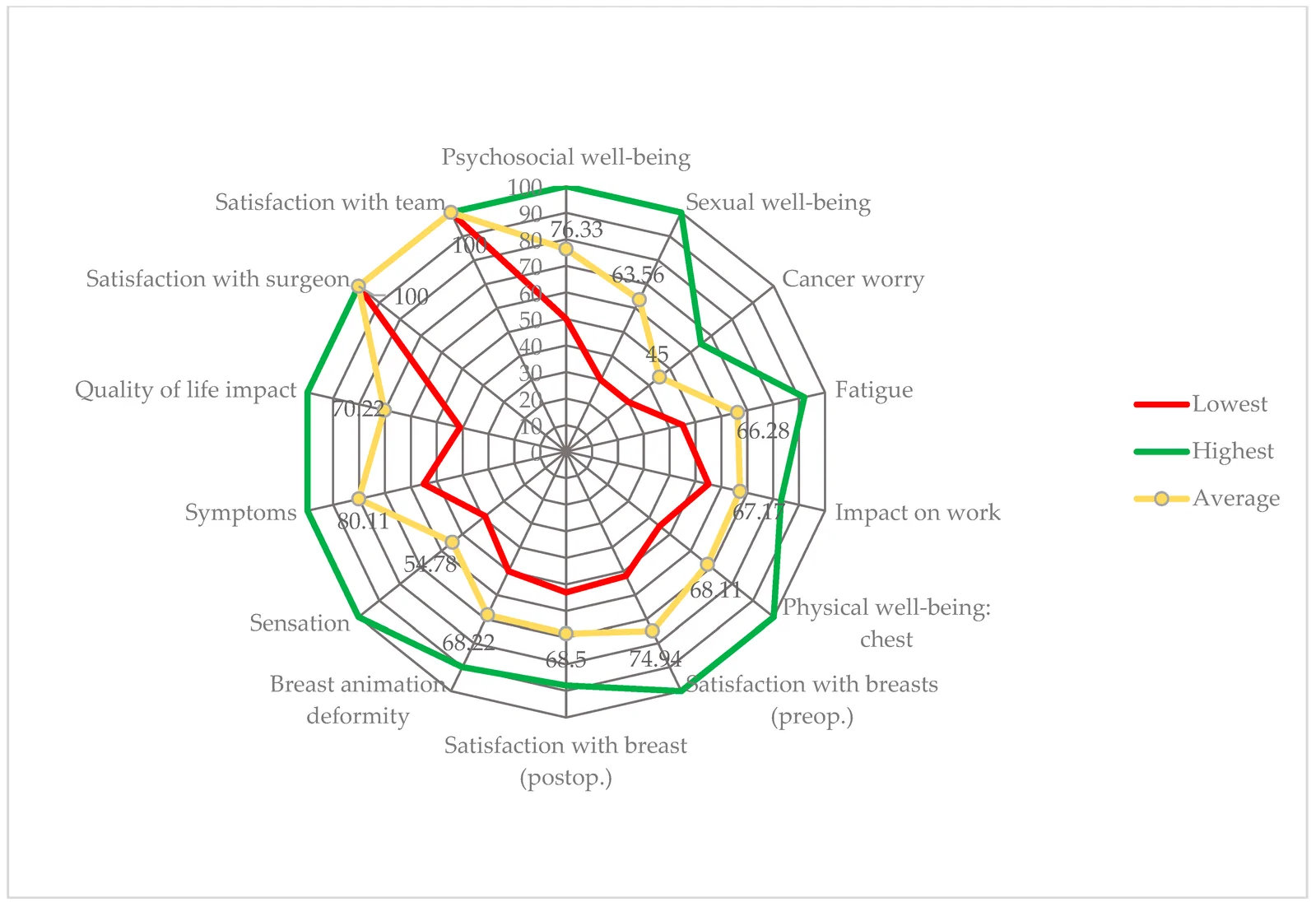
BREAST-Q results.

The BREAST-Q questionnaire was distributed to all patients at 12 months postoperatively. The results of the survey are illustrated in Figure 10.

The average score was 66.94 (range: 31–100), the highest values being reported for the Psychosocial Well-being and Symptoms scales (76.33 and 80.11, respectively), apart from Satisfaction with the breast surgeons and medical team, which attained maximum scores. Postmenopausal women reported higher Psychosocial and Sexual well-being. The patients who reported lower Fatigue scores had larger implant volumes compared to those who performed better, and this category included the LD reconstruction patient and all those who opted for simultaneous HBSO. Better Impact on work scores were observed for patients with smaller breast sizes and shorter operative times, as well as stage I and II cancer at diagnosis. The lowest mean scores were observed in the Cancer worry (45, range: 31–64) and Breast sensation (54.77, range: 38–100) categories. Cancer worry was more severe in premenopausal women and those with more advanced stage at diagnosis, as well as in patients who had simultaneous contralateral operations or HBSO. Regarding satisfaction with implants and the presence of contour irregularities, most patients (61.11%) were satisfied with the results and five patients reported complete satisfaction.

Patients with higher Physical well-being scores had better skin coverage (BTCC type 3), while most of those who scored lower were younger or had also had radiotherapy. Improved Sensation correlated with BTCC type 3. Superior Symptoms scores were achieved by patients having lower BMI, more advanced cancer and history of chemotherapy, who also had higher postoperative pain levels.

The Harris and Ueda scores were provided by two independent surgeons who did not actively participate in the management of the patients. Excellent result rates reached 77.78% with the Harris score and 33.33% with the Ueda score, while 22.22% and 44.4%, respectively, achieved good aesthetic outcomes. A fair evaluation according to Ueda’s standards was given in 22.22% of patients, but none of the patients were deemed to have poor reconstructive results.

## 4. Discussion

Endoscopic breast surgery was performed during the 1980s for the excision of benign breast masses and the early 1990s to release capsular contracture secondary to augmentation [41,42]. However, the premises for its application in oncologic breast surgery were only established at the end of the century, after nipple-sparing mastectomy was accepted as a standard, oncologically safe and cosmetically favorable technique for patients with early-stage breast cancer and those requesting risk-reducing surgery, the latter being greatly popularized by Hartmann et al. along with increasing awareness of the effects of BRCA1 and 2 mutations [29,43,44].

Retrospectively, it appears that Asian surgeons have been able to gain abundant experience with video-assisted breast surgery owing to their patients’ relatively lower breast size compared to the Western female population [45]. Nevertheless, as Kitamura et al. imply, another major driving factor might have been the desire to provide a minimally invasive approach with aesthetically pleasing outcomes to small-breasted women diagnosed with early-stage breast cancer [12].

Sae-Lim et al. have proven that minimal-access mastectomy with reconstruction (including both endoscopic and robotic-assisted procedures) results in diminished blood loss and lower incidence of severe complications compared to conventional NSM for large breasts, as well as comparable hospital stay durations for all breast sizes [21]. Minimal-access breast surgery is linked to reduced postoperative immune suppression and faster immune cell function recovery compared to open procedures, similarly to the immune impact of thoracoscopic/laparoscopic procedures as opposed to their open counterparts, as demonstrated in a recent study by Jiang et al. [35,46].

Our selection algorithm was generally based on the inclusion and exclusion criteria utilized by other studies, but we did not exclude older patients, smokers and patients with previous breast surgery or chest radiotherapy [8,21,22,23,24,36,37]. Other variations regard tumor size (<3.5 cm), staging (up to IIIA), axillary lymph node status and multicentricity/multifocality [21,29,36]. Mok et al. point out that neoadjuvant therapy is a valid means of extending the indication to patients initially considered unsuitable for VAM by downsizing the tumor [22].

The initial incision was tailored to the diameter of the single-port device to avoid gas leakage and was subsequently enlarged (if necessary) to remove the gland and insert the implant [35]. In order to maintain small incision lengths, we employed a funnel for “no-touch” prosthesis insertion, which was also reported to reduce the risk of capsular contracture, a complication of prepectoral implant placement [47]. Thus, the incision lengths in our case series align with the values reported in literature for axillary placement (between 2 and 5 cm) [14,29,45] and fall below the values reported for latero-pectoral, mid-axillary line or lateral mammary placement (between 4 and 6 cm) [8,12,16,36]. According to the literature, both sentinel lymph node dissection and lymphadenectomy can be performed through the same axillary incision under direct vision, and our case series also included two patients in whom the VAM was preceded by sentinel lymph node excision [36,45,47]. Lymphadenectomy might indeed require enlarging the incision, but endoscopic lymph node dissection techniques capable of preserving the initial incision length have also been described, employing either a vein retractor or carbon dioxide insufflation with optional preemptive liposuction [45].

No periareolar incisions were necessary, which resulted in the absence of NAC loss or partial necrosis. Dissection in the proximity of the NAC should be performed using classic scissors or minimal coagulation, since it is widely agreed that minimal thermal damage to the NAC is essential to preserve its color, sensitivity and viability [29,36].

Two distinct methods for developing a proper working space during VAM have been described: insufflation, which requires single-port or multiple trocar placement, and retraction, necessitating wound protection devices in addition to the retractors themselves or external traction systems [12,20,22,23,48]. Intraoperative frozen section assessment is necessary for the base of the NAC and strongly recommended for the superficial margin of the tumor [8,29]. Few studies utilize the dissection sequence applied in our case series—subcutaneous dissection followed by prepectoral dissection, which would be preferable to avoid the difficult manipulation of the breast caused by its separation from the posterior fascia [8]. However, several authors prioritize prepectoral dissection, counting on the Cooper ligaments’ ability to suspend the gland to maintain the working space, and there is evidence that this approach diminishes blood loss and shortens operative time [23,28,29,49,50,51,52]. This order is chosen especially when subpectoral dissection is also included in the equation—notably, the term “reverse sequence endoscopic-assisted mastectomy” defines VAM starting with subpectoral dissection for implant pocket development [53,54].

Prepectoral breast reconstruction was carried out in all cases, and several other authors support this approach [17,25,29]. Minimal disruption of the breast footprint, enhanced preservation of skin flap vascularity, remoteness of the incision from maximum tension zones on the breast mound and absence of future radiotherapy to the reconstructed breast diminish the need to provide additional support and coverage for the implant, which are the main reasons for subpectoral implant placement [8,23,55,56,57]. Polyurethane-coated implants are particularly suitable for direct-to-implant breast reconstruction in the preprectoral plane due to decreased capsular contracture rates and stable position [30,58]. Excellent postoperative satisfaction scores are obtained in patients with adequate and uniform skin flap thickness (BTCC types 2 and 3), which can be more precisely controlled during procedures having enhanced visualization such as endoscopic and robotic mastectomy [30,31,59]. Most of the implants used in our study were polyurethane-coated (66.67%), and the average volume of 313.89 mL was similar to the values presented in other studies for both endoscopic (304.3 ± 70.1 mL) and robotic (312.5 mL) breast surgery [15,17]. Meshes can be used in video-assisted breast surgery to stabilize the implant in prepectoral breast reconstruction and support the heavier implants required for reconstruction in large-breasted patients (“the parachute method”) [29,60].

Minimally invasive breast surgery is associated with fewer overall and grade III complications compared to open procedures, ranging from 1.1% to 18% according to Mok et al.’s review [22]. The most frequently reported were NAC or skin necrosis (3.4–11.6%, due to inadequate incision placement and failure to protect the primary pedicles during subcutaneous dissection), delayed healing or wound edge necrosis (7.01–14.3%), skin burns, seroma (4–11%) and infections resulting in capsular contracture or implant exposure [37,61,62,63].

Endoscopic-assisted breast surgery achieves low local, as well as regional and distant, recurrence rates and high overall survival, with no significant difference compared to open NSM [12,13,22,63]. Indeed, the latter is associated with local recurrence rates of 1% to 12% during a mean follow-up interval of 10 to 76 months across various studies, while endoscopic-assisted NSM exhibits local recurrence rates of 1.8% to 5.3%, with the exception of Ito et al., who reported no local recurrences in a cohort with mean follow-up up of 51.2 months [22,37]. Multicentric disease, extensive lymphovascular invasion, high tumor grade and certain immunohistochemistry phenotypes (triple negative, luminal B, Her2 overexpression, absence of estrogen receptors and high Ki67) are among other risk factors associated with a significant local recurrence rate in the NSM literature and should be pondered when establishing the indication for VAM, as well [63].

A particularity of our case series is the inclusion of patients whose breasts were previously operated on, which was treated as an exclusion criterion in previous studies. The first patient with this characteristic enrolled in our study was a stage IIIB patient with a previous mastectomy, who eventually developed in situ lobular carcinoma on the contralateral breast, treated by quadrantectomy, and who opted for mastectomy and bilateral reconstruction.

The other two patients benefited from symmetrization by reductional mastopexy at the time of breast reconstruction and at a future date requested risk-reduction mastectomy, being BRCA2 mutation carriers, as well. However, the diffuse bleeding of the scar tissue and its relative rigidity decreased the quality of visualization and raised the difficulty of the dissection, necessitating an amplification of the insufflation pressure to 12 mm Hg [8,15,29]. Both patients developed intraoperative hypercapnia, which translated into headache, dizziness, nausea and prolonged postoperative pain during the first 24–48 h after surgery. We therefore suggest caution when modifying the insufflation pressure.

Zhang et al. reported that endoscopic-assisted NSM with reconstruction resulted in larger drainage volume compared to the open procedure, which in turn caused larger intraoperative blood loss [23]. The mean interval required to allow safe drain removal was longer compared to patients undergoing skin-sparing mastectomy with reconstruction in our hospital as well. The same authors state that the increased costs incumbent on the utilization of endoscopic instruments was balanced by the reduced hospitalization inherent to minimally invasive surgery, resulting in only 101.18 USD higher hospitalization costs for VAM [23]. Notably, the medical costs associated with VAM in our case series were much lower than those reported by Lai et al. for both conventional and reverse sequence endoscopic NSM (6404.2 USD and 5063.4 USD on average, respectively) and half the costs estimated for robotic NSM (between 10,000 and 12,000 USD) [59,62].

Several factors can explain the reluctance to embrace video-assisted breast surgery, starting from the high cost of initial installation and overcoming the learning curve to the increased operative time [14,23,24]. Moreover, plastic surgeons often express concern about the limited size of the implants that can be inserted or flaps that can be anastomosed through such small incisions, ultimately compromising the aesthetic outcomes of the procedure in the name of minimal invasiveness [14,29].

The long duration of endoscopic-assisted procedures is a consequence of the difficulty of securing an adequate surgical field and maintaining a consistent optical window around the curvature of the breast mound or after the superficial dissociation of the gland from the subcutaneous tissue, in addition to dealing with rigid instrumentation and frequent collision in a restricted working space, due to the limitations for conventional endoscopic instruments [4,8,14,23,24]. Although many studies have obtained significantly longer operative times for endoscopic compared to conventional breast surgery (30 to 60 min), this aspect did not influence postoperative complication rates and was reduced after surpassing the learning curve [22].

Our study is the proof that borrowing the skills and knowledge of laparoscopic surgeons and starting VAM in a medical center equipped according to the current standards in laparoscopic surgery—including single-port devices and more flexible and ergonomic instruments such as the articulating vessel sealer and cutter, which simplifies dissection and exploration in the mammary gland—can alleviate the learning curve and make up for some of the technical inconveniences that have so far only been surmounted by robotic surgery.

In addition to being less expensive than robotic breast surgery, VAM takes advantage of the surgeon’s tactile sensation in controlling flap thickness and often proves to be more time-efficient due to less elaborate instrument setting and a faster learning curve [13,24]. According to Lai et al., a significant decrease in total operative time is expected from the 10th endoscopic-assisted mastectomy with reconstruction, whereas over 15 surgeries are necessary for the robotic-assisted equivalent [13,62].

Most studies that present operative times report subpectoral implant placement and therefore contain a supplementary step with which we have not experimented so far in video-assisted breast surgery. In our case series, the total operative times were largely influenced by the reconstructive step, which varied according to the procedures performed on the contralateral breast. Only three cases in our series had no such procedures, and their operative times (210, 200 and 125 min, respectively) are situated at the lower extreme of the intervals reported for VAM with subpectoral reconstruction by Chung et al. (408.9 ± 87.4 min for the conventional, prepectoral-to-retropectoral sequence and 279.4 ± 83.9 min for the reverse sequence) and Zhang et al. (a minimum of 133 min with an average of 193.71 ± 28.75 min for Zhang et al.’s optimized method and 324.80 ± 66.39 min for the conventional one) and much lower compared to Franceschini et al. (an average of 347 min using the skin-lift method and including axillary management) [23,50,63]. A recently published French case series presenting prepectoral breast reconstruction after VAM reports a total surgical time of 125.1 ± 30.6 min [25]. This supports the time efficiency of our strategy to offer minimally invasive RRM to patients scheduled for delayed or second-stage breast reconstruction during the same anesthetic session by having two surgeons working on different breasts and represents a novel way of applying the co-surgeon model in oncoplastic surgery [63].

The limitations of the present study lie in the small sample size limiting the possibility of proper statistical assessment. Another point of concern is the relatively short follow-up interval, which precluded an evaluation of the oncologic safety of the procedure and the development of late complications, including surface deformity and capsular contracture. A multicenter study might bring to light other particularities of the learning curve and a greater variety of patients having specific demands and breast/tumor characteristics to be initially addressed on a case-by-case basis and then centralized for further statistical analysis.

The success of new procedures, including patient (and surgeon) satisfaction, is dependent upon diligent patient selection and adaptation of existing knowledge and techniques to each case’s particularities, which should be achieved by combining the surgical team’s experience with the evidence provided by well-defined standards and guidelines.

## 5. Conclusions

The preliminary results show that video-assisted mastectomy is an innovative, time- and cost-effective technique capable of creating high quality and reliable skin flaps for immediate implant-based breast reconstruction. By combining the benefits of minimally invasive techniques with oncological safety, this method supports aesthetically favorable outcomes. As such, it has the potential to significantly contribute to the improvement of postoperative psychological well-being and overall quality of life in patients with early-stage breast cancer and may represent a valuable advancement in the field of breast reconstruction.

## Figures and Tables

**Table 1:** Oncologic characteristics.

Oncologic Characteristic	Subtype	Number of Patients	Percentage
Tumor location	Contralateral breast (operated)	15	83.33%
	Ipsilateral breast (TM)	3	16.67%
Breast cancer type	Ductal carcinoma	12	66.67%
	Lobular carcinoma	5	27.78%
	Lobular carcinoma (in situ)	1	11.11%
Grading	G1	6	33.33%
	G2	10	55.56%
	G3	12	11.11%
Staging	IA	4	22.22%
	IB	2	11.11%
	IIA	4	22.22%
	IIB	3	16.67%
	IIIA	5	27.78%
Chemotherapy	Pre-VAM	13	72.22%
Radiotherapy	Pre-VAM	10	55.56%
Hormonal therapy	Pre-/post-VAM	18	100%
Herceptin therapy	Pre-VAM	5	27.78%
Post-mastectomy breast volume	None	5	27.78%
	Expander/implant (mean volume)	268.13 mL (range: 175–450 mL)	
First mastectomy to VAM interval	(mean)	21.75 months (range: 6–60 months)	
Previous ipsilateral breast surgery	Quadrantectomy	2	11.11%
	Breast reduction/mastopexy	4	22.22%

**Table 2:** Procedure details.

Patient	Incision (cm)	Implant Type	Implant Volume (mL)	Mastectomy Volume (mL)	Difference (mL)	Skin Perfusion (After Implant Placement)	Concomitant Procedure
1	4	P	330	157.87	0	Good	EI
2	3.5	M	350	115	25	Good	EI
3	4	M	450	422	20	Suboptimal	EI
4	3	P	350	298.02	50	Good	LD
5	4,5	P	295	245.56	5 ^1^	Good	None
6	3	P	220	147.98	0	Suboptimal	IR + HBSO
7	3.5	P	320	256.67	0	Good	None
8	2.5	P	195	113.68	20	Good	None
9	3	M	315	315	0	Suboptimal	EI
10	3	P	320	267.84	0	Good	EI
11	3	P	340	246.23	0	Suboptimal	IR + HBSO
12	2.5P	P	220	138.4	20	Suboptimal	IR
13	3	M	265	225.43	15	Good	None
14	2.5	P	195	150.12	0	Good	IR
15	3	P	320	304.83	10 ^1^	Good	None
16	3.5	M	380	324.55	0	Good	EI
17	3	M	340	288.61	15	Suboptimal	EI
18	3	P	280	233.41	0	Good	HBSO

^1^ indicates unilateral reconstruction, difference based on contralateral breast preoperative estimated volume. Abbreviations: P: polyurethane-coated; M: microtextured; EI: expander-to-implant exchange; HBSO: hysterectomy and bilateral salpingo-oophorectomy; IR: implant reconstruction; LD: latissimus dorsi and implant reconstruction.

## Data Availability

The original contributions presented in this study are included in the article. Further inquiries can be directed to the corresponding authors.

## Abbreviations

The following abbreviations are used in this manuscript:

NAC	nipple-areolar complex
BCS	breast conserving surgery
NSM	nipple-sparing mastectomy
BTCC	breast tissue coverage classification
VAM	video-assisted mastectomy
RRM	risk-reducing video-assisted mastectomy
TM	therapeutic video-assisted mastectomy
VAS	visual assessment scale
BMI	body mass index
EI	expander-to-implant exchange
HBSO	hysterectomy and bilateral salpingo-oophorectomy
IR	implant reconstruction (without contralateral procedure)
LD	latissimus dorsi flap and implant reconstruction
USD	United States dollars

## References

[1] Stevens L.A., McGrath M.H., Druss R.G., Kister S.J., Gump F.E., Forde K.A. (1984). The psychological impact of immediate breast reconstruction for women with early breast cancer. Plast. Reconstr. Surg..

[2] Roy N., Downes M.H., Ibelli T., Amakiri U.O., Li T., Tebha S.S., Balija T.M., Schnur J.B., Montgomery G.H., Henderson P.W. (2024). The psychological impacts of post-mastectomy breast reconstruction: A systematic review. Ann. Breast Surg..

[3] Brandberg Y., Sandelin K., Erikson S., Jurell G., Liljegren A., Lindblom A., Lindén A., von Wachenfeldt A., Wickman M., Arver B. (2008). Psychological reactions, quality of life, and body image after bilateral prophylactic mastectomy in women at high risk for breast cancer: A prospective 1-year follow-up study. J. Clin. Oncol..

[4] Toesca A., Peradze N., Galimberti V., Manconi A., Intra M., Gentilini O., Sances D., Negri D., Veronesi G., Rietjens M. (2017). Robotic nipple-sparing mastectomy and immediate breast reconstruction with implant: First report of surgical technique. Ann. Surg..

[5] Satteson E.S., Brown B.J., Nahabedian M.Y. (2017). Nipple-areolar complex reconstruction and patient satisfaction: A systematic review and meta-analysis. Gland Surg..

[6] Shiah E., Laikhter E., Comer C.D., Manstein S.M., Bustos V.P., Bain P.A., Lee B.T., Lin S.J. (2022). Neurotization in innervated breast reconstruction: A systematic review of techniques and outcomes. J. Plast. Reconstr. Aesthetic Surg..

[7] Kerwin L.Y., El Tal A.K., Stiff M.A., Fakhouri T.M. (2014). Scar prevention and remodeling: A review of the medical, surgical, topical and light treatment approaches. Int. J. Dermatol..

[8] Tukenmez M., Ozden B.C., Agcaoglu O., Kecer M., Ozmen V., Muslumanoglu M., Igci A. (2014). Videoendoscopic single-port nipple-sparing mastectomy and immediate reconstruction. J. Laparoendosc. Adv. Surg. Tech. A.

[9] Friedlander L.D., Sundin J., Bakshandeh N. (1995). Endoscopy mastectomy and breast reconstruction: Endoscopic breast surgery. Aesthetic Plast. Surg..

[10] Ingram D. (2008). Is it time for breast cancer surgeons to embrace endoscopic-assisted mastectomy?. ANZ J. Surg..

[11] Ho W.S., Ying S.Y., Chan A.C. (2002). Endoscopic-assisted subcutaneous mastectomy and axillary dissection with immediate mammary prosthesis reconstruction for early breast cancer. Surg. Endosc. Other Interv. Tech..

[12] Kitamura K., Ishida M., Inoue H., Kinoshita J., Hashizume M., Suginachi K. (2002). Early results of an endoscope-assisted subcutaneous mastectomy and reconstruction for breast cancer. Sugery.

[13] Yu D.Y., Lee T.Y., Kim D.W., Chang Y.W., Son G.S., Lee H.Y. (2025). Preliminary experience and learning curve of endoscopic nipple-areolar-complex sparing total mastectomy: A single-center retrospective study. PLoS ONE.

[14] Satake T., Narui K., Muto M., Ishikawa T., Maegawa J. (2018). Endoscopic nipple-sparing mastectomy with immediate multistage fat grafting for total breast reconstruction: A new combination for minimal scar breast cancer surgery. Plast. Reconstr. Surg..

[15] Lai H.W., Lin S.L., Chen S.T., Kuok K.M., Chen S.L., Lin Y.L., Chen D.R., Kuo S.J. (2018). Hybrid technique for nipple-sparing mastectomy: Technique, preliminary results, and patient-reported cosmetic outcome from preliminary 50 procedures. Ann. Surg. Oncol..

[16] Fan L.J., Jiang J., Yang X.H., Zhang Y., Li X.G., Chen X.C., Zhong L. (2009). A prospective study comparing endoscopic subcutaneous mastectomy plus immediate reconstruction with implants and breast conserving surgery for breast cancer. Chin. Med. J..

[17] Sarfati B., Struk S., Leymarie N., Honart J.F., Alkhashnam H., Tran de Fremicourt K., Conversano A., Rimareix F., Simon M., Michiels S. (2018). Robotic prophylactic nipple-sparing mastectomy with immediate prosthetic breast reconstruction: A prospective study. Ann. Surg. Oncol..

[18] Sakamoto N., Fukuma E., Higa K., Ozaki S., Sakamoto M., Abe S., Kurihara T., Tozaki M. (2009). Early results of an endoscopic nipple-sparing mastectomy for breast cancer. Ann. Surg. Oncol..

[19] Park K.U., Cha C., Pozzi G., Kang Y.J., Gregorc V., Sapino A., Gazzetta G., Marrazzo E., Toesca A. (2019). Robot-assisted nipple sparing mastectomy with immediate breast reconstruction: An initial experience. Sci. Rep..

[20] Wang X., Wan X., Li L., Liu X., Meng R., Sun X., Xiao C. (2023). Trans-axillary single port insufflation technique-assisted endoscopic surgery for breast diseases: Clinic experience, cosmetic outcome and oncologic result. Front. Oncol..

[21] Sae-Lim C., Lai H.W., Lin S.L., Huang H.I., Chen S.T., Chen D.R. (2024). Is minimal-accessed (endoscopic- or robotic-assisted) nipple-sparing mastectomy contraindicated for large breasts?. Eur. J. Surg. Oncol..

[22] Mok C.W., Lai H.W. (2019). Endoscopic-assisted surgery in the management of breast cancer: 20 years review of trend, techniques and outcomes. Breast.

[23] Zhang S., Xie Y., Liang F., Wang Y., Wen N., Zhou J., Feng Y., Liu X., Lv Q., Du Z. (2022). Video-assisted transaxillary nipple-sparing mastectomy and immediate implant-based breast reconstruction: A novel and promising method. Aesthetic Plast. Surg..

[24] Rathat G., Herlin C., Bonnel C., Captier G., Duraes M. (2019). Endoscopic nipple-sparing mastectomy with immediate prepectoral implant-based reconstruction: A case report. Am. J. Case Rep..

[25] Rathat G., Chaumette M., Fontaine V., Rebel L., Pissarra J., Duflos C., Duraes M. (2025). Endoscopic prophylactic nipple-sparing mastectomy: First French survey of 10 patients. J. Gynecol. Obstet. Hum. Reprod..

[26] Nigro L.C., Blanchet N.P. (2017). Animation deformity in postmastectomy implant-based reconstruction. Plast. Reconstr. Surg. Glob. Open.

[27] Xu X., Gao X., Pan C., Hou J., Zhang L., Lin S. (2024). Postoperative outcomes of minimally invasive versus conventional nipple-sparing mastectomy with prosthesis breast reconstruction in breast cancer: A meta-analysis. J. Robot. Surg..

[28] Duncan A.M., Al Youha S., Joukhadar N., Konder R., Stecco C., Wheelock M.E. (2022). Anatomy of the breast fascial system: A systematic review of the literature. Plast. Reconstr. Surg..

[29] Huang Z., Li Z., Zhong X., Yang Q., Wei L., Li D., Li H. (2024). Endoscopic bilateral nipple-sparing mastectomy via a single axillary incision with immediate pre-pectoral implant-based breast reconstruction. J. Vis. Exp..

[30] Salgarello M., Pagliara D., Barone Adesi L., Visconti G., Wild J.B., Matey P. (2021). Direct to implant breast reconstruction with prepectoralmicropolyurethane foam-coated implant: Analysis of patient satisfaction. Clin. Breast Cancer.

[31] Rancati A., Angrigiani C., Hammond D., Nava M., Gonzalez E., Rostagno R., Gercovich G. (2016). Preoperative digital mammography imaging in conservative mastectomy and immediate reconstruction. Gland Surg..

[32] Kaufman C.S. (2019). Increasing role of oncoplastic surgery for breast cancer. Curr. Oncol. Rep..

[33] Avino A., Gheoca-Mutu D.E., Răducu L., Nedelea S.A., Jecan C.R., Lascar I. (2021). Patient-reported quality of life 3 months after breast reconstruction. Chirurgia (Bucur).

[34] Gheoca D.E., Avino A., Răducu L., Marina C.N., Ștefan M.C., Tomescu L.F., Tulin A.D., Jecan C.R. (2021). 6 years of breast reconstruction in one center—An objective analysis. Chirurgia (Bucur).

[35] Yamashita K., Shimizu K. (2006). Endoscopic video-assisted breast surgery: Procedures and short-term results. J. Nippon Med. Sch..

[36] Chen Y., Xu J., Hong S., Xu S. (2024). A challenging surgical technique: Single-port endoscopic-assisted radical mastectomy in retrograde way and immediate reconstruction using prosthesis implantation. Transl. Cancer Res..

[37] Lai H.W., Chen S.T., Chen D.R., Chen S.L., Chang T.W., Kuo S.J., Kuo Y.L., Hung C.S. (2016). Current trends in and indications for endoscopy-assisted breast surgery for breast cancer: Results from a six-year study conducted by the taiwan endoscopic breast surgery cooperative group. PLoS ONE.

[38] Pusic A.L., Klassen A.F., Scott A.M., Klok J.A., Cordeiro P.G., Cano S.J. (2009). Development of a new patient-reported outcome measure for breast surgery: The BREAST-Q. Plast. Reconstr. Surg..

[39] Harris J.R., Levene M.B., Svensson G. (1979). Analyses of cosmetic results following primary radiation therapy for stages I and II carcinoma of the breast. Int. J. Radiat. Oncol. Biol. Phys..

[40] Ueda S., Tamaki Y., Yano K., Okishiro N., Yanagisawa T., Imasato M., Shimazu K., Kim S.J., Miyoshi Y., Tanji Y. (2008). Cosmetic outcome and patient satisfaction after skin-sparing mastectomy for breast cancer with immediate reconstruction of the breast. Surgery.

[41] Kitamura K., Hashizume M., Sugimachi K., Kataoka A., Ohno S., Kuwano H., Maehara Y. (1998). Early experience of endoscopic extirpation of benign breast tumors via an extra-mammary incision. Am. J. Surg..

[42] Kompatscher P. (1992). Endoscopic capsulotomy of capsular contracture after breast augmentation: A very challenging therapeutic approach. Plast. Reconstr. Surg..

[43] De La Cruz L., Moody A.M., Tappy E.E., Blankenship S.A., Hecht E.M. (2015). Overall survival, disease-free survival, local recurrence, and nipple-areolar recurrence in the setting of nipple-sparing mastectomy: A meta-analysis and systematic review. Ann. Surg. Oncol..

[44] Hartmann L.C., Sellers T.A., Schaid D.J., Frank T.S., Soderberg C.L., Sitta D.L., Frost M.H., Grant C.S., Donohue J.H., Woods J.E. (2001). Efficacy of bilateral prophylactic mastectomy in BRCA1 and BRCA2 gene mutation carriers. J. Natl. Cancer Inst..

[45] Owaki T., Kijima Y., Yoshinaka H., Hirata M., Okumura H., Ishigami S., Nerome Y., Takezaki T., Natsugoe S. (2015). Present status of endoscopic mastectomy for breast cancer. World J. Clin. Oncol..

[46] Jiang Q., Liao J., Tan J., Hu H. (2024). Comparison of minimal access and open breast surgery: A propensity score-matched study on postoperative immune function in breast cancer. World J. Surg. Oncol..

[47] Sakamoto N., Fukuma E., Teraoka K., Hoshi K. (2016). Local recurrence following treatment for breast cancer with an endoscopic nipple-sparing mastectomy. Breast Cancer.

[48] Flugstad N.A., Pozner J.N., Baxter R.A., Creasman C., Egrari S., Martin S., Messa C.A., Oliva A., Schlesinger S.L., Kortesis B.G. (2016). Does implant insertion with a funnel decrease capsular contracture? A preliminary report. Aesthet. Surg. J..

[49] Qiu M., Liang F., Xie Y., Yang H., Zhang Q., Zhong J., Dai H., Du Z. (2024). ASO author reflections: A novel technique of transaxillary reverse-sequence endoscopic nipple-sparing mastectomy and direct-to-implant prepectoral breast reconstruction. Ann. Surg. Oncol..

[50] Wang Y., Wu J.X., Guan S. (2017). A technique of endoscopic nipple-sparing mastectomy for breast cancer. JSLS.

[51] Rehnke R.D., Groening R.M., Van Buskirk E.R., Clarke J.M. (2018). Anatomy of the superficial fascia system of the breast: A comprehensive theory of breast fascial anatomy. Plast. Reconstr. Surg..

[52] Chung K., Xie Y., Liang F., Qiu M., Yang H., Zhang Q., Dai H., Du Z. (2024). Reverse-sequence endoscopic nipple-sparing mastectomy with immediate implant-based breast reconstruction: An improvement of conventional minimal access breast surgery. Front. Oncol..

[53] Yamaguchi S., Asao T., Uchida N., Yanagita Y., Saito K., Yamaki S., Kuwano H. (2008). Endoscopy-assisted subcutaneous mastectomy and immediate breast reconstruction for breast cancer: Advantage of the posterior approach. Int. Surg..

[54] Ostapenko E., Nixdorf L., Devyatko Y., Exner R., Wimmer K., Fitzal F. (2023). Prepectoral versus subpectoral implant-based breast reconstruction: A systemic review and meta-analysis. Ann. Surg. Oncol..

[55] Salgarello M., Barone Adesi L., Macrì G., Visconti G. (2023). When to consider prepectoral implant conversion after subpectoral implant breast reconstruction and how to plan it. Aesthet. Surg. J..

[56] Correia-Pinto J.M., Andresen C., Barbosa J.P., Poleri F., Casimiro R., Gonçalves D., Baptista D., Coelho G., Cunha C., Costa H. (2024). Impact of polyurethane versus acellular dermal matrix coating on prepectoral reconstruction outcomes: Interface does matter. J. Plast. Reconstr. Aesthetic Surg..

[57] Ito K., Kanai T., Gomi K., Watanabe T., Ito T., Komatsu A., Fujita T., Amano J. (2008). Endoscopic-assisted skin-sparing mastectomy combined with sentinel node biopsy. ANZ J. Surg..

[58] Lai H.W., Mok C.W., Chang Y.T., Chen D.R., Kuo S.J., Chen S.T. (2020). Endoscopic assisted breast conserving surgery for breast cancer: Clinical outcome, learning curve, and patient reported aesthetic results from preliminary 100 procedures. Eur. J. Surg. Oncol..

[59] Leff D.R., Vashisht R., Yongue G., Keshtgar M., Yang G.Z., Darzi A. (2011). Endoscopic breast surgery: Where are we now and what might the future hold for video-assisted breast surgery?. Breast Cancer Res. Treat..

[60] Endara M., Chen D., Verma K., Nahabedian M.Y., Spear S.L. (2013). Breast reconstruction following nipple-sparing mastectomy: A systematic review of the literature with pooled analysis. Plast. Reconstr. Surg..

[61] Lai H.W., Wang C.C., Lai Y.C., Chen C.J., Lin S.L., Chen S.T., Lin Y.J., Chen D.R., Kuo S.J. (2019). The learning curve of robotic nipple sparing mastectomy for breast cancer: An analysis of consecutive 39 procedures with cumulative sum plot. Eur. J. Surg. Oncol..

[62] Franceschini G., Visconti G., Garganese G., Barone-Adesi L., Di Leone A., Sanchez A.M., Terribile D., Salgarello M., Masetti R. (2020). Nipple-sparing mastectomy combined with endoscopic immediate reconstruction via axillary incision for breast cancer: A preliminary experience of an innovative technique. Breast J..

[63] Mallory M.A., Losk K., Camuso K., Caterson S., Nimbkar S., Golshan M. (2016). Does “two is better than one” apply to surgeons? Comparing single-surgeon versus co-surgeon bilateral mastectomies. Ann. Surg. Oncol..

